# Supplementary material for: Identification of photoperiod-induced specific miRNAs in the adrenal glands of Sunite sheep (Ovis aries)
Source: Front Vet Sci. 2022 Jul 22;9:888207. doi: 10.3389/fvets.2022.888207 (PMC9354845; doi:10.3389/fvets.2022.888207)
Supplement: Supplementary file 1 [file Data_Sheet_1.ZIP › Supplementary file/miRDeep2 file.zip!/miRDeep2 file/Novel_Structure/SPLP42A1/Novel_575.pdf]

Provisional ID	:	NC_019469.2_18634
Score total	:	1.1
Score for star read(s)	:	-1.3
Score for read counts	:	0
Score for mfe	:	0.8
Score for randfold	:	1.6
Score for cons. seed	:	
Total read count	:	24
Mature read count	:	24
Loop read count	:	0
Star read count	:	0

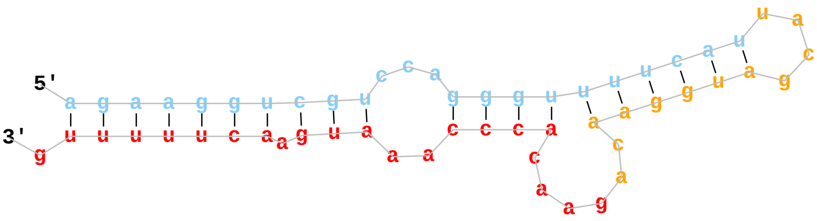

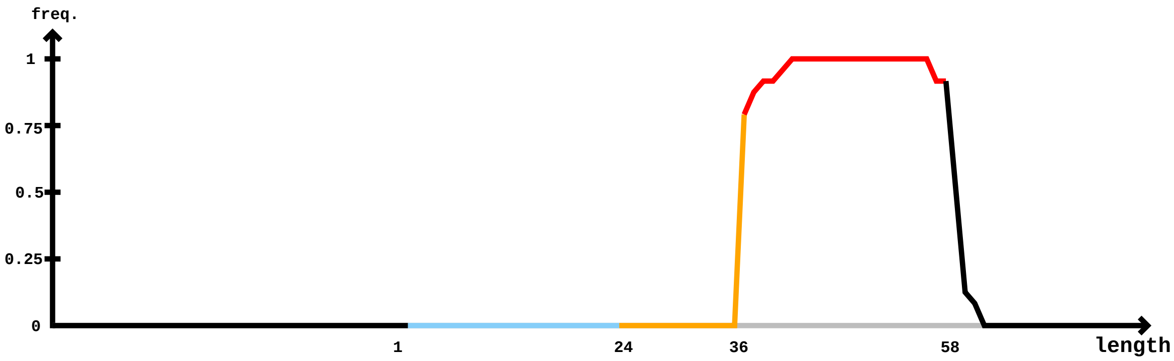


```
                                        Star                                 Mature
5'- agccacuggaacccccucuguuaucggguuggccaagaaggucguccaggguuuucauuacgauggaacagaacacccaaaugaacuuuuuggcaggcccaauaccaaucac   -3'    exp
    .......((.....))..((.(((.(((((.(((((((((.((((...((((((((((....))))))......))))..)))).))))))))).))))).))).)).....    reads      mm         sample
    ......................................................................gaacacccaaaugaacuuuuug....................    1          0          seq
    ......................................................................gaaAacccaaaugaacuuuuug....................    18         1          seq
    .......................................................................aaGacccaaaugaacuuuu......................    2          1          seq
    ........................................................................aGacccaaaugaacuuuuuggc..................    1          1          seq
    ..........................................................................acccaaaCgaacuuuuuggca.................    1          1          seq
    ...........................................................................cccGaaugaacuuuuuggca.................    1          1          seq
```
